# Intraoperative Cardiac Arrest and Mortality in Trauma Patients. A 14-Yr Survey from a Brazilian Tertiary Teaching Hospital

**DOI:** 10.1371/journal.pone.0090125

**Published:** 2014-02-27

**Authors:** Marcelo T. O. Carlucci, José R. C. Braz, Paulo do Nascimento, Lidia R. de Carvalho, Yara M. M. Castiglia, Leandro G. Braz

**Affiliations:** 1 Department of Anesthesiology, Botucatu Medical School, UNESP – Univ Estadual Paulista, Botucatu, São Paulo, Brazil; 2 Department of Biostatistics, Institute of Biosciences, UNESP – Univ Estadual Paulista, Botucatu, São Paulo, Brazil; University of South Florida, United States of America

## Abstract

**Background:**

Little information on the factors influencing intraoperative cardiac arrest and its outcomes in trauma patients is available. This survey evaluated the associated factors and outcomes of intraoperative cardiac arrest in trauma patients in a Brazilian teaching hospital between 1996 and 2009.

**Methods:**

Cardiac arrest during anesthesia in trauma patients was identified from an anesthesia database. The data collected included patient demographics, ASA physical status classification, anesthesia provider information, type of surgery, surgical areas and outcome. All intraoperative cardiac arrests and deaths in trauma patients were reviewed and grouped by associated factors and also analyzed as totally anesthesia-related, partially anesthesia-related, totally surgery-related or totally trauma patient condition-related.

**Findings:**

Fifty–one cardiac arrests and 42 deaths occurred during anesthesia in trauma patients. They were associated with male patients (*P*<0.001) and young adults (18–35 years) (*P* = 0.04) with ASA physical status IV or V (*P*<0.001) undergoing gastroenterological or multiclinical surgeries (*P*<0.001). Motor vehicle crashes and violence were the main causes of trauma (*P*<0.001). Uncontrolled hemorrhage or head injury were the most significant associated factors of intraoperative cardiac arrest and mortality (*P*<0.001). All cardiac arrests and deaths reported were totally related to trauma patient condition.

**Conclusions:**

Intraoperative cardiac arrest and mortality incidence was highest in male trauma patients at a younger age with poor clinical condition, mainly related to uncontrolled hemorrhage and head injury, resulted from motor vehicle accidents and violence.

## Introduction

Trauma is a major concern in public health worldwide causing 5 million deaths per year and accounting for one of every 10 deaths [1]. It is the leading cause of death among people under 40 years of age [2]. In the USA, trauma is responsible for more adolescent deaths than other causes combined [2]. In Brazil, trauma was the third-leading cause of death (12.4%) in 2005, exceeded only by cancer (14.7%) and cardiovascular diseases (28.3%) [3]. Motor vehicle crashes, vehicular accidents involving pedestrians, and gunshot wounds are the most common causes of death among Brazilians aged 15–39 years, and the third-leading cause of death among those in the 10–49 year age group [3]. Mortality due to trauma is projected to increase by 40% by the year 2030 (particularly, mortality due to traffic accidents, which is associated with economic growth in low- and middle-income countries) [4].

Anesthesia induces physiological changes that might lead to morbidity and mortality. However, due to improved monitoring techniques and intensive patient care, perioperative cardiac arrest and death rates have declined over the past two decades [5], [6]. Although a few studies on perioperative [7], [8] and intraoperative [9] cardiac arrest have indeed included trauma patients, they did not evaluate trauma exclusively. As a result, the factors influencing intraoperative cardiac arrest and its outcomes in trauma patients remain unclear. A recent study did not find any difference between the outcomes of trauma and nontrauma patients who experienced intraoperative cardiac arrest, with 16% and 14% of survival rate, respectively [10].

This study examined all cases of cardiac arrest occurring in trauma patients during anesthesia over a 14-year period in a Brazilian teaching hospital, to assess the associated factors and outcomes.

## Methods

The project was approved by the Institutional Ethics Committee (Ref: 2108/2006) that waived the requirement for written informed consent. This survey reviewed all events of intraoperative cardiac arrest and mortality in patients with trauma reported to occur from January 1, 1996 to December 31, 2009 in the Botucatu Medical School Hospital, UNESP, Brazil. This 450-bed public tertiary referral teaching hospital, located close to two important São Paulo State highways, includes a trauma center and intensive care units for children (10 beds) and adults (25 beds). It provides multi-specialty surgical care in an area with more than 2 million inhabitants, with about 7,000 surgeries being performed on patients of all ages every year. Anesthesia care is provided by full-time faculty anesthesiologists, staff anesthesiologists and residents.

All trauma patients were examined by an anesthesiologist immediately before emergency and urgent surgical procedures according to the interventions and protocols for Advanced Cardiovascular Life Support (ACLS) guidelines. Basic monitoring in the operating room (OR) during regional and neuraxial anesthesia consisted of continuous ECG display, automatic non-invasive blood pressure and pulse oximetry. For general anesthesia, core temperature, capnography, delivered oxygen and anesthetic vapor concentration, and ventilation parameters were also measured. Critical patients were also subjected to direct invasive blood pressure monitoring and central venous access.

### Data Collection

Cardiac arrest and death events occurring during anesthesia in the OR were identified from the hospital anesthesia database created on the basis of quality-assessment forms that are part of the mandatory documentation of every anesthetic procedure. The forms, under the responsibility of the staff anesthesiologists, collected information on the place and time of the procedure, patient demographics, surgical procedures (elective, urgent or emergency surgery), surgical area, American Society of Anesthesiologists (ASA) physical status classification, type of anesthesia (general, regional or monitored anesthesia care in critically ill patients), and included a 95-item checklist of airway, respiratory, cardiocirculatory, neurological, renal and miscellaneous events related to OR. When regional and general anesthesia were combined, general anesthesia was considered to be the technique used.

Cardiac arrest was defined as the cessation of cardiac mechanical activity with loss of effective circulation determined by the absence of a palpable central pulse. Only the events occurring from the moment that the anesthesia team took over the patient at the OR to the moment the patient was transferred to surgical ward or intensive care unit were included in the analysis. The analysis was restricted to those patients experiencing intraoperative cardiac arrest within 24 hours after trauma. The anesthetist responsible for each case was asked to review the case and provide a written summary for peer review. This procedure eliminated the possibility of incomplete data-collection forms.

The medical and anesthesia records, written summary, and necropsy report (when applicable) were reviewed by the Anesthesia Cardiac Arrest Study Commission, which consisted of three faculty members of the Department of Anesthesiology (JRCB, PNJr, and LGB). The cardiac arrest and death were classified as: (i) totally anesthesia-related (anesthesia was the only or the major contributing factor); (ii) partially anesthesia-related (the patient’s disease/condition or surgical procedure were contributing factors, but anesthesia represented an additional factor); (iii) totally surgery-related; or (iv) totally related to the trauma patient condition. Cardiac arrest and death events were jointly analyzed by all three commission members. There was unanimity on the causes of most cardiac arrests and deaths. Disagreements among the three members were resolved by discussion and agreement or consensus was reached in all cases when at least two of the three members agreed on the cause.

The associated factors, listed as patients characteristics, surgical area, type of anesthesia, mechanism of trauma and its main injury were also analyzed.

### Statistical Analysis

The 95% confidence interval (95% CI) for event proportions was calculated. The chi-squared test was used to compare the proportion of cardiac arrest and death in trauma patients according to gender. The Tukey-type test for multiple comparisons among proportions [11] with the Bonferroni correction were used to compare cardiac arrest proportion as a function of trauma patient age, ASA physical status, surgical area, anesthetic technique and mechanism of trauma and injuries. The power of the test was calculated as at least 80% and at most 95%. The Statistic Package for Social Sciences software (version 6.0; SPSS Inc., Chicago, IL, USA) was used for the statistical analysis. Significance level was set at *P* value <0.05.

## Results

During the 14-year study period, 90,909 patients with and without trauma were anesthetized. Fifty-one intraoperative cardiac arrests with high mortality (42 deaths - 82,3%) occurred in trauma patients, corresponding to 18.21% and 23.20% of the total number of intraoperative cardiac arrests (280) and deaths (181), respectively. All of the cardiac arrest and death events in trauma patients took place in emergency surgeries.

The majority of cardiac arrest in trauma patients were observed in adults aged 18–35 years (78.4% of all intraoperative cardiac arrests that occurred in this age group, *P = *0.04) (Table 1); males (the ratio of male to female cardiac arrest patients was 4∶1; *P*<0.001); ASA physical status IV or poorer (*P*<0.001) undergoing gastroenterological or multiclinical surgeries (*P*<0.001; Table 2); and undergoing general anesthesia (*P*<0.001; Table 3). Motor vehicle accidents and violence were the main causes of trauma in these patients (*P*<0.001; Table 4). Considering all causes of cardiac arrest and death among anesthetized patients, trauma was the second most important cause of cardiac arrest and the most important cause of death; sepsis with multiple organ failure was the most important cause of cardiac arrest and the second most important cause of death.

**Table 1:** Intraoperative cardiac arrest proportions in trauma patients by the number of all cardiac arrests in all patients, grouped by age.

	All cardiac arrests	Cardiac arrests in trauma patients (n = 51)		
	(n = 280)	n	Proportion* (%)	95% CI
**Age group (yr)**				
0–17	58	2	3.44c	0.00–8.14
18–35	37	29	78.37a	65.11–91.64
36–50	42	13	30.95b	16.97–44.93
51–64	62	1	1.61c	0.00–4.75
65–79	62	5	8.06c	1.29–14.84
≥80	19	1	5.26c	0.00–15.30

CI: confidence interval;

*Proportions followed by different letters are significantly different (*P* = 0.04).

**Table 2:** Intraoperative cardiac arrest and mortality proportions in trauma patients, grouped by gender, ASA physical status and surgical area.

	Cardiac arrests (n = 51)			Deaths (n = 42)		
	n	Proportion* (%)	95% CI	n	Proportion* (%)	95% CI
**Gender**						
Male	41	80.4a	69.50–89.29	34	80.95a	69.08–92.83
Female	10	19.6b	8.71–30.50	8	19.04b	7.17–30.92
**ASA physical status**						
I	0	0.0c	0.00–0.00	0	0.0c	0.00–0.00
II	0	0.0c	0.00–0.00	0	0.0c	0.00–0.00
III	2	3.92c	0.00–9.25	2	4.76c	0.00–11.20
IV	13	25.49b	13.53–37.45	12	28.57b	14.91–42.23
V	36	70.58a	58.08–88.09	28	66.66a	52.41–80.92
**Surgical area**						
Gastroenterological	18	35.29a	22.18–48.41	16	38.09a	23.41–52.78
Multiclinical	17	33.33a	20.40–46.27	14	33.33a	19.08–47.59
Thoracic	5	9.80b	1.64–17.97	3	7.14b	0.00–14.93
Neurosurgery	4	7.84b	0.46–15.22	3	7.14b	0.00–14.93
Vascular	2	3.92b	0.00–9.25	2	4.76b	0.00–11.20
Orthopedic	2	3.92b	0.00–9.25	2	4.76b	0.00–11.20
Pediatrics	2	3.92b	0.00–9.25	1	2.38b	0.00–6.99
Cardiac	1	1.96b	0.00–5.77	1	2.38b	0.00–6.99

CI: confidence interval;

*Proportions followed by different letters are significantly different (*P*<0.001).

**Table 3:** Intraoperative cardiac arrest and mortality proportions in trauma patients, grouped by anesthetic technique.

	Cardiac arrests (n = 51)			Deaths (n = 41)		
Anesthetic technique	n	Proportion* (%)	95% CI	n	Proportion* (%)	95% CI
General anesthesia	32	62.74a	49.5–76.0	25	59.52a	46.04–75.91
Regional anesthesia						
- Epidural/spinal	2	3.92c	0.00–9.25	2	4.76b	0.00–11.47
- Plexus block	0	0.0c	0.00–0.00	0	0.0b	0.00–0.00
Other#	17	33.33b	20.40–46.27	15	35.71ab	21.4–51.33

CI: confidence interval;

# Monitored anesthesia and supportive care in patients with an ASA V physical status;

*Proportions followed by different letters are significantly different (*P*<0.001).

**Table 4:** Intraoperative cardiac arrest proportions, grouped by trauma cause.

	Cardiac arrests		
Trauma cause	n	Proportion* (%)	95% CI
**Motor vehicle**	**32**	**62.74a**	49.48–76.01
Crashes	28	54.90	41.25–68.56
Accidents involving pedestrians	4	7.84	0.46–15.22
**Violence**	**15**	**29.41b**	16.91–41.92
Stab wounds	6	11.76	2.92–20.61
Gunshot wounds	5	9.81	1.64–17.97
Beatings	4	7.84	0.46–15.22
**Others**	**4**	**7.84c**	0.46–15.22
Roof fall	2	3.92	0.00–9.25
Electrocution	1	1.96	0.00–5.77
Fall from a cow	1	1.96	0.00–5.77

CI: confidence interval;

*Proportions followed by different letters are significantly different (*P*<0.001).

All intraoperative cardiac arrests and deaths were totally related to patient’s clinical condition. Severe injury with uncontrolled hemorrhage and head injury were the main associated factors in these patients (P<0.001; Table 5). Mortality rate was high (at least >66.7%) for all main mechanisms of injury. Death was mainly related to traumatic amputations, massive hepatic or splenic lacerations, multiple fractures, lung contusions or traumatic brain injury.

**Table 5:** Intraoperative cardiac arrest and death proportions in trauma patients, grouped by associated factor.

	Cardiac arrests (n = 51)			Deaths (n = 42)			Mortality
Causes	n	Proportion* (%)	95% CI	n	Proportion* (%)	95% CI	(%)
Abdominal hemorrhage	17	33.33a	20.40–46.27	14	33.33a	19.08–47.59	82.4
Head injury	8	15.68ab	5.71–25.67	6	14.28ab	3.70–24.87	75.0
Thoracic and abdominal hemorrhage	7	13.72ab	4.28–23.17	5	11.90ab	2.11–21.70	71.4
Thoracic hemorrhage	6	11.76ab	2.92–20.61	5	11.90ab	2.11–21.70	83.3
Abdominal and pelvic hemorrhage	4	7.84b	0.46–15.22	4	9.52b	0.65–18.40	100.0
Abdominal hemorrhage+head injury	3	5.88b	0.00–12.34	3	7.14b	0.00–14.93	100.0
Limbs hemorrhage	3	5.88b	0.00–12.34	2	4.76b	0.00–11.20	66.7
Pelvic hemorrhage	1	1.96b	0.00–5.77	1	2.38b	0.00–699	100.0
Bleeding from limbs+head injury	1	1.96b	0.00–5.77	1	2.38b	0.00–6.99	100.0
Lung embolism after leg fracture	1	1.96b	0.00–5.77	1	2.38b	0.00–6.99	100.0

IC: confidence interval;

*Proportions followed by different letters are significantly different (*P*<0.001).

## Discussion

This study provides detailed information on the associated factors and outcomes of intraoperative cardiac arrest associated with trauma in 51 patients who were anesthetized under current practices in a tertiary referral center over a 14-year period.

Patients who had intraoperative cardiac arrest after 24 hours of injury were excluded from this study because trauma may induce multiple organ failure and sepsis or systemic inflammatory response syndrome in these cases.

Perioperative and intraoperative cardiac arrests and mortality in trauma patients typically account for 20%–25% of all perioperative cardiac arrests and deaths [7]–[9], which is consistent with our findings. In our study, trauma was the second most important cause of cardiac arrest and the leading cause of mortality following cardiac arrest, with only 17.64% of the patients surviving. In a study, trauma was the most common cause of perioperative cardiac arrest and mortality, with 0% of the patients surviving [7]. The typically poor ASA physical status of the trauma patients (96% were ASA IV or ASA V) was certainly an important factor of intraoperative cardiac arrest and mortality in our study and underscores the severity of trauma in the patients who suffered a intraoperative cardiac arrest.

Trauma-related cardiac arrest is known to carry a poor survival prognosis [12], [13]. Approximately 50% of trauma deaths occur at the site of the trauma, and 20% in the first two hours after the trauma [14]. In a 10-year retrospective study of trauma patients receiving out-of-hospital cardiopulmonary resuscitation, 81.4% died outside the hospital or in the emergency department; and only 7.5% survived to hospital discharge [15]. Cardiopulmonary resuscitation was effective in only 18% of the trauma patients admitted to the emergency room with cardiac arrest [16], while 40% of trauma patients aged >20 years with cardiac arrest who were resuscitated and admitted to the intensive care unit survived to hospital discharge [13]. A study showed 84% of mortality in trauma patients who experienced intraoperative cardiac arrest [10], which is in accordance with our findings. The poor physical status of trauma patients in the OR poses a challenge to modern anesthesia and surgery. Uncontrolled hemorrhage remains the greatest threat, occurring in approximately 40%–60% of intraoperative deaths related to trauma [10], [17], [18].

Even though our data involved a long period of time and several new approaches have been suggested in emergency trauma care [19], our data is similar to those reported in the literature [7], [9], [10] and probably little would change if only the last five years had been analyzed. Prompt transportation to specialized trauma care and immediate management of life-threatening injuries are the first and main steps in trauma resuscitation [19]. Quick surgical control of bleeding is pivotal in trauma care. Specialized pre-hospital care associated with an emergency specialized hospital team has been provided in the area our data is related over the last 14 years. We believe that only educational strategies and prevention could potentially change these dramatic numbers.

The high incidence of intraoperative cardiac arrest in young men observed in this study reflects the increased susceptibility to trauma and violence seen in males [20]. Additionally, more than 2.5 million individuals aged 10 to 24 years die every year from external causes worldwide [2], [20], [21]. In 1930, external causes accounted for 3% of the deaths in Brazil. In 2005, it was 12.5% (72.4 per 100,000 inhabitants) with a rate of 122.5 per 100,000 for men and a much lower rate for women (22 per 100,000) [3]. In Brazil, homicide is the main cause of death by external causes in adolescents (41.7 per 100,000), followed by motor vehicle accidents (25.7 per 100,000). The incidence of suicides is much lower (5.1 per 100,000). Over the past decade, developing countries have lost a generation of young people to violence [22]. These data are in contrast to the results of a recent Brazilian survey that found a gradual improvement in health conditions and quality of life. Life expectancy in Brazil increased to 73.5 years in 2011 [23]. However, this figure is two or three years lower than it would be without premature mortality due to external causes [3].

There are differences in deaths from external causes between developed and developing countries. In England, intentional self-harm is the main cause of death from external causes among young males, followed by transportation accidents and falls. The incidence of homicide is lower [24]. A comparative study on the epidemiology of urban trauma deaths in the USA (Denver, Colorado) found that, between 1992 and 2002, there was a significant increase in the rate of transport-related and fall-related deaths, and a significant reduction in the rate of deaths from intentional injuries [25]. In this study, the three predominant causes of fatal injury were transportation accidents (43%), intentional self-harm (24%) and falls (20%).

Our findings show that patient condition resulting from external causes was the main associated factor of intraoperative cardiac arrest and death in trauma patients. Therefore, many high-risk surgeries in trauma patients are performed under general anesthesia rather than regional anesthesia. The absence of anesthesia-related cardiac arrest and death in trauma patients, as observed in our study, reinforces the safety improvement seen in anesthesia over the last two decades [5], [6].

This study should be interpreted in the context of the following limitations. First, study data were derived from adverse event reports completed by faculty members and residents. Underreporting is likely in this situation, even though form completion for each case is mandatory. To minimize such risk, the information was cross-checked with operating theatre records and hospital administration files. Second, it was not possible to precisely determine the incidence of intraoperative trauma cardiac arrest because we did not have information on the exact number of trauma patients submitted to anesthesia. Third, data were derived from a single institution. Practices peculiar to the institution may have influenced our findings and may not be fully generalisable to other healthcare settings. The development of multi-institutional anesthesia databases will not only provide useful insights into mechanisms of injury, but will also allow the development of strategies that can be applied universally [26], [27].

## Conclusions

The study identified 51 intraoperative cardiac arrests with high mortality in trauma patients, corresponding to approximately 20% of the total number of intraoperative cardiac arrests and deaths. Intraoperative cardiac arrest following trauma was prevalent in ASA physical status IV (or poorer) males at a younger age, undergoing gastroenterological or multiclinical surgery under general anesthesia. All of the cardiac arrest events were attributable to trauma patient conditions (uncontrolled hemorrhage and head injury) that resulted from motor vehicle accidents and violence. Analyzing intraoperative cardiac arrests in trauma patients may be instructive for developing prevention strategies.
